# Factors associated with risky driving behaviors for road traffic crashes among professional car drivers in Bahirdar city, northwest Ethiopia, 2016: a cross-sectional study

**DOI:** 10.1186/s12199-019-0772-1

**Published:** 2019-03-09

**Authors:** Tesfaye Hambisa Mekonnen, Yitayew Ashagrie Tesfaye, Haimanot Gebrehiwot Moges, Resom Berhe Gebremedin

**Affiliations:** 1 Department of Environmental and Occupational Health and Safety, Institute of Public Health, College of Medicine and Health Sciences, University of Gondar, P.O. Box 196, Gondar, Ethiopia; 2 Debre Tabor Zone Health Office, Debre Tabor, Amhara Regional State Ethiopia; 3 Department of Health promotion and education, Institute of Public Health, College of Medicine and Health Sciences, University of Gondar, Gondar, Ethiopia

**Keywords:** Risky driving behavior, Associated factors, Road traffic crashes, Drivers, Ethiopia

## Abstract

**Background:**

Road traffic injury is one of the persistent public health challenges in most regions of the world, representing substantial human and economic losses. Annually, about 1.25 million lives are lost, whereas 50 million suffer from road traffic injuries globally. It has been shown that over 60% of the reasons for traffic injuries are a risky driving behavior (RDB). Despite the problem’s pervasiveness, there is a paucity of information about level and factors influencing RDB among professional car drivers in Bahirdar city, northwest Ethiopia.

**Methods:**

An institution-based cross-sectional study was conducted from February to March 2016. A systematic random sampling technique was used to select 376 participants. A self-administered driver behavior questionnaire (DBQ) was used for data collection. We performed a binary logistic regression analysis to investigate the associations of variables. Potential confounders were controlled using a multivariable logistic regression model. We ascertained the significance at < 0.05 *p* value and evaluated strength of associations using crude odds ratios (COR) and adjusted odds ratios (AOR) with 95% confidence intervals (CI).

**Results:**

A total of 361 drivers participated (response rate, 96%). The mean age was 34 (standard deviations ± 7.97) years. The majority, 98.9% (*N* = 357), were males. The level of risky driving behavior and road traffic crashes were 79.4% (95% CI 75.92, 83.97) and 16.3% (95% CI 15.91, 24.84), respectively. Average monthly salary [AOR 2.04; 95% CI (1.23, 2.74)], driving experience [AOR 2.72; 95% CI (1.07, 6.89)], distance driven per year [AOR 2.06; 95% CI (1.13, 4.10)], and previous history of involvement in traffic crashes [AOR 2.30; 95% CI (1.15, 7.35)] were significantly associated with risky driving behavior.

**Conclusions:**

The study shows that risky driving behavior is common among professional car drivers in the study setting. Therefore, it is strongly advisable for policy makers and other stake holders to devise strategies that consider working conditions, like monthly salary and driving experiences. The study also suggests that it is often advisable to reduce the distance driven per year and learn from implications of previous history of involvement in traffic crashes.

## Background

Road traffic injury (RTI) is the persistent public health challenges in most regions of the world, comprising substantial human and economic losses. Every year, about 1.25 million people lose their lives, and nearly 50 million more are injured or disabled, whereas 1–3% of gross national product (GNP) is lost from RTI globally [1]. Literature report shows that road traffic injury is the eighth leading cause of death worldwide [2]. It also overruns costs of healthcare services and imposes an additional burden on public health efforts made to combat chronic and non-chronic diseases [2, 3].

Over 90% of the world’s fatalities on roads occurs in low and middle-income countries despite the low number of the world’s registered vehicles (48%) available in these regions [4, 5]. African regions, including Ethiopia, encounter with the highest road traffic fatality rate [6, 7]. In these regions, rapid urbanization and motorization account for much of the rise and the rise is aggravated due to lack of appropriate road engineering and injury prevention programs [8]. Moreover, road traffic injuries are often one of the neglected public health problems because in developing countries traffic injuries are usually perceived as diseases of development outcomes [9].

Road traffic accident in Ethiopia is a cause of significant losses of human and economic resources. The occurrence of traffic accidents is increasing in the country as the exposure to this risk increases with rapid motorization (without appropriate regulation), rapid population growth, and increase in the road network coupled with poor attitude and safety culture of road users [3]. There is also no clear road safety policy, strategy, or program in the country, despite pervasiveness of the extent and severity of road traffic accident to road transport. Reports demonstrate that about 12,140 fatal and 29,454 injury crashes occurred between July 2005 and June 2011 in Ethiopia [10]. According to that report, the fatal crashes involved 1070 drivers, 5702 passengers, and 7770 pedestrians, totaling 14,542 fatalities, an average of 1.2 road user fatalities per crash. The report also exhibited that the majorities of the crashes occur during daytime hours, involve males, and involve persons in the 18–50 age groups, and crashes frequently occur in mid-blocks or roadways. The predominant collision between motor vehicles and pedestrians was a rollover on a road tangent section and failing to observe the priority of pedestrians and speeding were the major causes of crashes attributed by police in accordance with that study. Compared to developed nations of the world, like the US, a study demonstrates that the fatality rate or injury crash is about 30 times higher in Ethiopia [11].

Previous reports indicated that the occurrence of road traffic injuries is influenced by several determinant factors. Scholars have categorized those factors into human errors, road environments, and vehicle conditions [4, 11–14]. The human factors, which are often expressed as human errors, play a significant role in traffic crash involvements [15, 16]. Risky driving is driver-related human error that prominently contributes to the occurrences of traffic injuries [17, 18]. Literature shows that about 95% of all the traffic crashes are due to driver-related dangerous behaviors [19].

Investigators have identified several factors that underpin the involvements in risky driving behaviors. For instance, studies have explored that drivers’ gender importantly influences the experiences of risky driving behavior [20, 21]. According to a previous report [22], risky driving is significantly higher for males than females. To the contrary, a finding from the US [20] indicated that adolescent and young adult females involved in the risky driving behavior due to the fact that they are often supposed to be prone to traffic violations. Age of drivers is another important factor that can affect involvement in risky driving behaviors [23, 24]. A report from Australia revealed that young and inexperienced drivers are at a high risk of involving in risky driving behavior [25].

Other studies also showed that driving experience [13, 15, 21, 25], socioeconomic context, and monthly salary [4], drivers’ level of education [9], distance (number of kilometers (km/miles driven per specific time) [26], smoking and alcohol drinking [19, 24, 27–30], drivers’ physical and mental abilities, and psychological factors, like personality type, emotions, and distraction by outside or inside stimuli [4, 23, 25, 31–33] are among the factors that predispose the experiences of risky driving behavior.

Despite ample research available in other parts of the world, we lacked adequate studies supporting policy decisions on varieties of determinant factors leading to risky driving behavior in Ethiopia, particularly in Bahirdar city. This study was, therefore, aimed to investigate the level and identify risk factors associated with risky driving behaviors among professional car drivers in Bahirdar city, northwest Ethiopia. Understanding the level and exploring various risk factors determining risky driving behavior could have of particular importance to initiate the designing of better preventative strategies.

## Methods

### Study design, area, and period

An institution-based cross-sectional study design was used in the current study. The study was conducted among professional car drivers working in public institutions in Bahirdar city, the capital of Amhara regional state, northwest Ethiopia, from February to March 2016. The city is about 565 km far from Addis Ababa, the capital of Ethiopia. According to the 2007 report of Central Statistical Agency (CSA) of Ethiopia [34], the total population of the city was 221,991 of whom 113,535 were women. There were about 58 public institutions employing more than 757 professional car drivers in the city at the time of data collection period [35]. The city is a regional city and is the leading center for tourist destinations, with a variety of attractions in the nearby Lake Tana and Blue Nile river. The tourist sites of monasteries within the islands of Lake Tana and Tis Issat Falls of the Blue Nile are among the top tourist attracting places [36]. The city is also interconnected to other cities of the country and therefore the flow of vehicles, including privately owned cars, three wheel vehicles/Bajaj, motors, bicycles (the most common way of traveling), city busses, mini-busses, taxies, public institutions/government vehicles, intercity bus service, and other commercial transportation vehicles in the city, is dramatically elevating from time to time, and road traffic crashes more frequently occur in the city than ever before [36]. Literature also reveals that the reported traffic accidents in Amhara Regional State constituted nearly 14% of all accidents and 26% of fatalities, which is quite high, compared to other regions, of which Bahir Dar city represents the major share [3]. At the same time, the population density of the city is also rapidly increasing and road users/pedestrians usually seem overcrowded. The infrastructural setups in the city, like construction of new asphalt roads and cobblestones are also increasing but are inconsistent compared to the number of populations and vehicles. Moreover, some roads are narrow and one way, others are without a pedestrian way, and the majorities of these roads are without appropriate traffic signs and signals.

### Sample size and sampling procedures

We employed the systematic sampling technique to select eligible participants. Epi info version 7 software was used to calculate the required sample. We computed a total of 376 participants using a single-population proportion formula. We assumed 66.6% proportion of risky driving behavior (RDB) based on previous study conducted in Mekele city [9], an absolute precision of 5%, and 95% confidence intervals to achieve adequate power during analysis. We systematically numbered 757 professional car drivers from 1 to *N* and took the interval *k* = *N*/*n* = 757/376 = 2 at every *k*^th^ or 2nd interval and finally reached 376 participants, where *N was* the study population, *n* = the calculated sample size and *k* = the interval size. After including an additional grant of 10% for non-respondents, we have attained 376 samples.

### Ethics approval

In this study, the ethical clearance was obtained from the Institutional Ethical Review Board (IERB) of the University of Gondar, College of Medicine and Health Sciences, Institute of Public Health (Reference No. EOHS/453/2008). All personal identifiers were removed and only aggregate data were used for analysis. Involvement in the study was based on the full consent of the person willingly accepting the participation.

### Operational definitions

#### Risky driving behavior

Risky driving behavior is behavior that involves experiencing at least one of the five risky driving behaviors in the past 12 months. The risky driving behavior includes speeding, drink-drive, unfastening of a seat belt, driving while feeling sleepy, and highway code violations [9].

#### Road traffic crash

Road traffic crash is a crash which is occurred when vehicles collided with other vehicles, with pedestrian and other stationary obstacles in the previous 12 months [9].

#### Speeding

Speeding is the distance which was driven above 40 km/hour in towns and above 80 km/hour outside in the last 12 months [33].

#### Drink-drive

Drink-drive is driving after drinking alcohol within 4 h in a specified period.

#### Chat chewer

Chat chewers are chewing an amount ≥ 25 g per chew per day for ≥ 1 day per week [37].

### Inclusion and exclusion criterion

#### Inclusion criterion

Professional drivers who had a driving professional license and had been working for at least 12 months prior to the study period were considered for inclusion.

#### Exclusion criteria

Professional car drivers who were absent from their work due to annual, sick, maternal, and family events, like death- and marriage-related leaves, during the data collection period were excluded.

#### Data collection tools and techniques

We collected data using a pre-tested and self-administered structured questionnaire. The standardized driver behavior questionnaire (DBQ) was used to assess the level of risky driving behavior. This questionnaire was first developed by Reason et al. [38] and considered appropriate for a self-report data collection technique [39]. The instrument was widely used to assess aspects of drivers’ behavior that reflect human error, lapses, and deliberate risky action; has been used in ranges of cultural settings; and was also tested and verified by the authors for its reliability and validity. We made a few modifications to the instrument in order to contextualize it to the country’s diverse situations. The questionnaire comprised of information about socio-demographic characteristics, driving environment exposure, and personal factors consisting of risky driving behavior, like speeding, driving while feeling sleepy, drink-drive, unfastening seat-belt, and high-way code violations.

#### Data quality control

To ensure quality of data, first and foremost, we focused on the data collection instrument. The modified DBQ questionnaire was developed in English and then translated to a local language, Amharic. The Amharic version was again translated back to English by language experts to check consistencies. Second, we trained five experienced data collectors and five supervisors for 2 days on issues relating to the instrument, data collection time, exclusion and inclusion criteria, and ethics approval/consent to participate. The principal investigator had also closely monitored both data collectors and supervisors daily. Third, we conducted a pretest at Debre Tabor zone public institutions on 19 drivers, which had characteristics nearly similar to the participants in the study. Based on the pretest results, we minimized contents of the questionnaire so as to optimize the participants’ time to fill the questions and modified some ambiguities. Finally, we checked the data for completeness before entry and analysis.

#### Variables and measurements

Risky driving behavior (RDB) is the dependent variable. We measured RDB using the self-reports of the participants on 4-point response options (always, often, sometimes, and never) by asking how often you…? (1) drove over 40 km/hour in towns and over 80 km/hour outside, (2) drove faster than the speed limit to get ahead of another vehicle, (3) drove after alcohol consumption, (4) drove without using a seat belt, (5) drove while feeling sleepy, (6) deliberately went through red lights, (7) raced with the other vehicles, (8) drove too close to the other vehicles, (9) crossed the pedestrian line while the pedestrians were waiting to cross, (10) overtook without a clear view and from the right-hand lane, (11) changed lanes without signaling to get ahead of the other vehicles, and (12) changed lanes or turned without using the rearview or side mirrors. For the purposes of logistic regression analysis, we categorized the 4-point response options to two: always, often, and sometimes = 1, presence of risky driving, while never = 0, no risky driving behavior. The detailed information on independent variables included socio-demographic variables, like sex, age, marital status, educational status, and average monthly salary [27]; driving environmental exposure factors, including driving-license level, driving experience, driven-hours per week, distance-driven per year (annual mileage) [33] and having previous history of crash involvement/crashes experienced before the study period; and personal factors, such as chat chewing, alcohol drinking, smoking, mood of driving, and level of risk perceptions.

### Data analysis

We used Epi-Info version 7 to enter and clean and Statistical Package for Social Sciences (SPSS) version 20 software to analyze data. We described the results using tables, percentages, means, and standard deviations. A multi-collinearity diagnosis between independent variables was checked using a variance inflation factor (VIF) at a cut off (VIF < 5) [40]. All the predictor variables included in the multivariate model were checked and found no evidence of collinearity. For example, the VIF for age and driving experience was 1.31; for driving hours per week and distance driven per year, it was 2.05; and for monthly salary and driving experience, VIF was 2.33. The correlations coefficients for the variables were also conducted using Pearson correlation coefficients (*r*). For example, the correlation coefficient between age and driving experience was (*r* = 0.013; *p* = 0.092), age and monthly salary (*r* = 0.012; *p* = 0.172), and age and driving hours per year (*r* = − 0.004; *p* = 0.123). A bivariate logistic regression model was performed separately for the independent variables to evaluate degrees of association with risky driving behavior (dependent variable). The independent variables with a < 0.2 *p* value [41] in this type of analysis were exported to a multivariable analysis to examine the effects of potential confounders. We selected the forward variable selection method in the multivariable logistic regression model since it was assumed to be relatively more conservative types. Hosmer and Lemeshow test was also checked for the goodness of fit for the model (*p* value > 0.05). We obtained significances of association at < 0.05 *p* value and evaluated strength using odds ratios with 95% confidence intervals.

## Results

### Drivers’ characteristics

A total of 361 drivers with a response rate of 96.0% participated in this study. The majority, 98.9% (*N* = 357), was males. The mean age was 34 (SD ± 7.97) years. More than half, 55.4% (*N* = 200), was married. Among the respondents, 56.0% (*N* = 202) indicated that they had attended secondary education (9–12 grades) and less (1–8 grades) and 44.0% (*N* = 159) above secondary education (college and/or university) (Table 1). Of the total participants, 35.7% (*N* = 129) had 1 to 4 years and 33% (*N* = 119) above 10 years of driving experiences. About less than half, 40.7% (*N* = 24), mentioned that the reasons for the accident involvements were speeding, 16.9% (*N* = 10) not giving priority to pedestrians, 15.2% (*N* = 9) road defects, 13.5% (*N* = 8) pedestrian defects, 8.5% (*N* = 5) vehicle defects, and 5.1% (*N* = 3) driving under fatigue. A high proportion, 72.6% (*N* = 262), of the respondents reported that driving fast made them happy, and a few respondents, 9.7% (*N* = 35), said they felt happy when people appreciated them for speeding. About 59.3% (*N* = 214) of the respondents pointed out that they had good levels of risk perception.

**Table 1:** Sociodemographic characteristics of drivers in Bahirdar city, Ethiopia, 2016 (*N* = 361)

Variables	Frequency	Percent (%)
Sex		
Male	357	98.9
Female	4	1.1
Age group		
≤ 29 years	137	38
≥ 30 years	224	62
Marital status		
Married	200	55.4
Single	147	40.7
Divorced/widowed	14	3.9
Educational status		
Secondary education and less	202	56
Above secondary	159	44
Monthly salary		
≤ 2000 ETB	216	59.9
> 2000 ETB	145	40.1

*ETB* Ethiopian Birr (currency), *N* number

### Prevalence of risky driving behavior and road traffic injury

The level of risky driving behavior in the previous 12 months was 79.5% (*N* = 287) (95% CI 75.9, 83.9). The prevalence of self-report road traffic crashes in the last 12 months was 16% (*N* = 59). A high proportion, 79.2% (*N* = 286), of the respondents indicated that they deliberately violated red lights. The majority, 71.2% (*N* = 257), demonstrated that they had driven over the prescribed distance limit (40 km/h in and over 80 km/h outside towns), while 63.4% (*N* = 229) showed that they had been involved in risky driving behavior due to racing with other vehicles. The least proportion of risky driving behavior described was changing lanes or turn without using rearview or side mirrors, 19.9% (*N* = 72) (Table 2).

**Table 2:** Risky driving behaviors among professional car drivers in Bahirdar city, Ethiopia, 2016

Risky driving behaviors in the last 12 months (*N* = 361)	Percent (%)	Mean (SD)
Deliberately going through red lights	79.2	3.11 (0.548)
Drive over 40 km/h in towns and over 80 km/h outside	71.2	2.91 (0.927)
Racing with other vehicles	63.4	3.13 (0.849)
Drive faster than the speed limit to get ahead of another vehicle	60.4	3.32 (0.617)
Driving too close to other vehicles	56.8	3.4 (0.569)
Driving without using a seat belt	52.3	3.35 (0.712)
Crossing pedestrian line while the pedestrians waiting to cross	50.7	3.45 (0.58)
Overtaking without a clear view and from the right hand lane	37.7	3.56 (0.661)
Change lanes without signaling to get ahead of other vehicles	32.4	3.63 (0.582)
Driving while feeling sleepy	31.9	3.67 (0.506)
Driving after alcohol consumption	27.4	3.71 (0.482)
Change lanes or turn without using rearview or side mirrors	19.9	3.78 (0.484)
At least one of the above	48.4	

*N* number, *SD* standard deviations,

*Note*: Likert scale scores *1 = always to 4 = never*

### Factors associated with risky driving behavior

In a bivariate logistic regression model, age, average monthly salary, driving experience, driving hours per week, distance driven per year, and previous crash involvement were the variables identified as significant factors of risky driving behavior.

In the multivariable logistic regressions analysis, average monthly salary, driving years of experience, distance driven per year, and the previous car crash involvement remained the important predictors of risky driving.

The result of multivariable logistic regression analysis demonstrated that respondents with a relatively high average monthly salary had 2.04 times more chance of having a risky driving behavior compared to drivers with the lower average monthly salary [AOR 2.04; 95% CI (1.23,2.74)]. The drivers whose driving experience was 1–4 years were 2.72 times more likely to engage in a risky driving behavior than the drivers whose driving experience was > 10 years [AOR 2.72; 95% CI (1.07, 6.89)].

The odds of having a risky driving behavior increased by a factor of 2.06 for those who had driven over 20,000 km than those who had driven less than 10,000 km per year [AOR 2.06;95% CI (1.13, 4.10)]. The drivers who had experienced road traffic accidents in the previous 12 months were 2.30 times more likely to commit risky driving behavior than those who had not experienced road traffic accidents in the same period [AOR 2.30; 95% CI (1.15,7.35)](Table 3).

**Table 3:** Factors associated with RDB among drivers in Bahirdar city, Ethiopia, 2016 (N = 361)

Variables	Risky driving behavior			
	Yes	No	COR (95% CI)	AOR (95% CI)
	*N* (%)	*N* (%)		
Age of driver				
≤ 29 years	123 (34.1)	14 (3.9)	3.14 (0.79, 6.68)	2.75 (0.41, 3.90)
≥ 30 years	165 (45.7)	59 (16.3)	1	1
Monthly salary			7770	
≤ 2000 ETB	185 (51.2)	31 (8.6)	1	1
> 2000 ETB	103 (28.5)	42 (11.6)	2.43 (1.48, 3.54)	2.04 (1.23, 2.74)**
Driving experience				
1–4 years	115 (31.8)	14 (3.9)	4.04 (2.04, 7.86)	2.72 (1.07, 6.90)**
5–10 years	93 (25.8)	20 (5.5)	2.27 (1.22, 4.20)	1.94 (0.96, 3.93)
> 10 years	80 (22.2)	39 (10.8)	1	1
Driving hours per week				
< 10 h	70 (19.4)	12 (3.3)	1	1
10–20 h	76 (21.1)	25 (7.0)	0.52 (0.24, 1.12) *	0.54 (0.23, 1.24)
> 20 h	142 (39.3)	36 (10.0)	0.68 (0.33, 1.38)	0.57 (0.25, 1.30)
Distance driven per year			1560	
< 10,000 km	118 (32.7)	22 (6.1)	1	1
10,000–20,000 km	110 (30.5)	25 (6.9)	1.91 (0.74, 2.75)	1.82 (0.91, 4.12)
> 20,000 km	60 (16.6)	26 (7.2)	2.32 (0.97, 3.52)	2.06 (1.13, 4.10)**
Previous history of traffic crash involvement				
Yes	53 (14.7)	6 (1.7)	2.52 (1.40, 6.11)	2.30 (1.15, 7.35)**
No	235 (65.1)	67 (18.5)	1	1

1$ USA = 27.5 ETB

*Significant in bivariate analysis only, **significant both in bivariate and multivariate logistic regression analysis

*AOR* adjusted odds ratio, *COR* crude odds ratios, *ETB* Ethiopian Birr (currency), *N* number; *RDB* Risky driving behavior

## Discussion

This study employed an institution-based cross-sectional design to investigate the level and identify diverse factors affecting the occurrences of risky driving behaviors among professional car drivers in Bahirdar city, Ethiopia. Our investigation showed that the proportion of participants who were involved in risky driving behaviors in the previous 12 months was 79.4% (95% CI: 75.92, 83.97). This result was higher than that of a previous report in Mekele city (66.6%) [9]. This might be due to the increasing levels of risky driving behaviors in the country from time to time. Our result was also higher than those of studies conducted in the UK, 17.8 and 13.6% (at different times) [24]. This could be explained by discrepancies in drivers’ observance of traffic rules and variations in national traffic policy enforcement across the countries. The level of risky driving behavior was, on the other hand, lower than that of a study done in the New Zealand (90%) [42]. This discrepancy might be because of differences in study samples and target population involved in the studies.

The magnitude of self-report road traffic crashes in the past 12 months was 16.3%. This was lower than previous reports from Mekele, 26.4% [27], 47% [43], and 62%, [44] Arba Minch (2 studies), Ethiopia. These variations could be due to differences in sample size, methods of data collections, and reference time frame, for example, in the Mekele study, the prevalence data were that of the previous 3 years (preceding the investigation). Besides, data for the Arba Minch work were gathered from the road traffic accident (RTA) trauma victims presented to the city hospital.

We found that monthly salary was considerably associated with risky driving behaviors. Drivers with a higher average monthly salary were four times more likely to have committed risky driving behavior as compared to drivers earning a lower average monthly salary. This result was in line with those of studies done in Mekele, Ethiopia [9], and Ghana [17]. A possible explanation might be that drivers with a higher monthly salary might drive faster than the limit prescribed because they might perceive time for their travel could affect their income generating desires. Another possible suggestion might be that they probably engage in drink-drive as they are supposed to be capable of frequently spending money on drinking.

Our finding demonstrated that drivers’ experience markedly associated with risky driving behavior. Previous researches reported comparable findings [13, 25, 45]. Accordingly, drivers with lower driving experience since their first driving license were more likely involved in risky driving behaviors compared to those with longer years of driving experiences. This might be due to the fact that inexperienced divers usually involve in driving faster than the recommended limits, on the one hand, and, on the other hand, they overestimate their driving skills and underestimate the potential risks of involving in high-speed driving behavior. This implies that good risk perception and adherence to safe driving behavior could be acquired through ample years of vehicle driving. Recent studies indicated that obedience to speed rules has possible protective capacity of involving in risky behaviors [45, 46]. Contrary to the current finding, however, an investigation result [47] showed that drivers with fewer years of driving experience demonstrated positive attitude towards obedience to speed rules than drivers with longer years of driving experiences. According to this study, as drivers get more experienced, their self-confidence increases and they likely engage in risky driving behaviors. The possible reasons for the inconsistent results could be due to differences in study participants.

In this study, the other significant association established with RDB was the number of kilometers driven per year. The drivers who drove relatively longer distances in the previous years were more likely engaged in the risky driving behaviors than those who drove shorter distances. This result was supported by studies in the Czech Republic [26], Turkey [45], and Iran [16]. A possible reason might be that drivers who drive longer distances per year may experience more occasions to commit traffic violations since they are highly likely to encounter with a higher exposure and spend more time on the road without necessary rest breaks. Without the reasonable rest breaks, driving is a profession recognized to cause chronic fatigue to drivers, leading to some kind of risky driving.

Our analysis has also shown that having a previous history of road traffic crash involvement in the year prior to this investigation period was a determinant factor for risky driving behavior. The drivers who had history of the road traffic crash involvement in the year before this study were 2.90 times more likely to engage in risky driving behavior compared to the drivers who had no crash history involvement. This finding corroborated with other studies [17, 42]. The drivers who had a history of traffic crash involvements in the past probably develop the ability to deliberately take risks and adapt to the persistent risky behavior, viewing it as a normal and are less likely to exercise self-control [21]. Such individuals might also become aggressive in order to avoid fears relating to the negative consequences of engaging in the risky driving behaviors. This explanation has been given by a previous research [45]. Further explanation may be that drivers who had the history of traffic crash experiences in the past might more repeatedly involve in both speeding and negligence of the traffic rules which are commonly deemed to be measures of risky behavior. A study has also provided a similar explanation [48]. However, it is imperative to note that beyond our interpretations, there is also a possibility that one is more careful in driving if the driver has an experience of a previous car crash.

This paper is not without some limitations. First, the assessment was based on the drivers’ self-report that may involve a recall bias, leading to under-reporting of the cases. Second, a social desirability bias might also occur because people usually tend to over-report the interests of investigators. But, we established a year time frame to minimize recall bias and used the valid and reliable DBQ questionnaire that was believed to lower the social desirability response. Third, the current study employed cross-sectional study design and hence, it is difficult to conclude the statistical inferences for the associations between risky driving behaviors and the factors influencing its occurrences due to the expected deficit temporal relationships. Fourth, while translating the assessment instrument from English to Amharic (the local language), the original meaning of the instrument may have been affected due to some potential technical errors. However, the translation was done by language professionals, fluent in English and Amharic; thus, the potential mistakes due to translation were kept minimal. Finally, since the current study only focused on professional car drivers of a specific sector, it might be uncertain to generalize the results of the study to car drivers of others sectors. Future investigations, therefore, would better incorporate professional car drivers of other sectors with a strong study designs, like longitudinal studies.

On the other hand, our study would contribute considerable inputs to the public health policies and practices. First, involvement in the risky driving behaviors more likely increases the occurrences of traffic injuries. The people getting injured due to the risky driving behaviors of a single driver might lose their incalculable lives, and others may encounter with permanent and temporary disabilities, which in turn affect the quality of life. Second, while injuries are taking place, they create an additional burden to the healthcare service utilization of a particular country. Therefore, policy makers and other stake holders hopefully benefit from our study to take initiatives that alleviate the risky driving behaviors leading to bottomless human and economic loses and the associated traffic injury tragedy.

## Conclusions

Risky driving behavior was high among drivers in Bahirdar city. Drivers’ average monthly salaries, lack of driving experience, distance driven per year, and having a history of traffic crash involvement prior to the investigation period were the factors considerably associated with the risky driving behaviors. Our study indicates that policy makers and other stake holders need to consider working conditions, like drivers’ monthly salary and driving experiences while devising interventions strategies. The study also suggests that it is quite advisable to reduce the distance driven per year and to learn from implications of a previous history of traffic crash involvement.

## Abbreviations

AOR: Adjusted odds ratio
CI: Confidence interval
DBQ: Driver behavior questionnaire
ETB: Ethiopian Birr
N: Number
OR: Odds ratios
RDB: Risky driving behavior
RTI: Road traffic injury
SD: Standard deviations
UK: United Kingdom
VIF: Variance inflation factor
WHO: World Health Organization
